# Rapid Microwave Synthesis, Characterization and Reactivity of Lithium Nitride Hydride, Li_4_NH

**DOI:** 10.3390/ma6115410

**Published:** 2013-11-21

**Authors:** Nuria Tapia-Ruiz, Natalie Sorbie, Nicolas Vaché, Tuan K. A. Hoang, Duncan H. Gregory

**Affiliations:** 1 WestCHEM, School of Chemistry, University of Glasgow, Glasgow G12 8QQ, UK; E-Mails: nuria.tapia@glasgow.ac.uk (N.T.-R.); natsor@chem.gla.ac.uk (N.S.); tuan.hoang@glasgow.ac.uk (T.K.A.H); 2 Ecole Nationale Supérieure de Chimie de Clermont-Ferrand, Université Blaise Pascal, BP 187, Aubière Cedex 63174, France; E-Mail: nicolas.vache@ensccf.fr

**Keywords:** nitride, hydride, structure, microwaves, synthesis, hydrogen storage, diffraction, thermal analysis, reactivity

## Abstract

Lithium nitride hydride, Li_4_NH, was synthesised from lithium nitride and lithium hydride over minute timescales, using microwave synthesis methods in the solid state for the first time. The structure of the microwave-synthesised powders was confirmed by powder X-ray diffraction [tetragonal space group *I*4_1_*/a*; *a* = 4.8864(1) Å, *c* = 9.9183(2) Å] and the nitride hydride reacts with moist air under ambient conditions to produce lithium hydroxide and subsequently lithium carbonate. Li_4_NH undergoes no dehydrogenation or decomposition [under Ar_(g)_] below 773 K. A tetragonal–cubic phase transition, however, occurs for the compound at *ca*. 770 K. The new high temperature (HT) phase adopts an *anti*-fluorite structure (space group *Fm*$\bar{3}$*m*; *a* = 4.9462(3) Å) with N^3−^ and H^−^ ions disordered on the 4*a* sites. Thermal treatment of Li_4_NH under nitrogen yields a stoichiometric mixture of lithium nitride and lithium imide (Li_3_N and Li_2_NH respectively).

## 1. Introduction

The Li–N–H system is a promising hydrogen storage candidate, with the ability to store 11.5 wt % of H_2_ reversibly [1]. This process occurs *via* two exothermic steps (Equations 1 and 2):

Li_3_N + H_2_ → Li_2_NH + LiH   ΔH = −165 kJ/mol H_2_ (1)

Li_2_NH + H_2_ → LiNH_2_ + LiH  ΔH = −44.5 kJ/mol H_2_ (2)

However, it has been demonstrated that the reaction pathway may be more complex than originally indicated. *In-situ* powder neutron diffraction (PND) [2,3] revealed the possibility of a reaction pathway involving the formation of the lithium nitride hydride, Li_4_NH [4,5] from lithium nitride in addition to the hydrogenated phase, lithium imide, Li_2_NH. At a low partial pressure of hydrogen, the formation of LiH appears to be suppressed, leading to the overall reaction shown in Equation (3). The dehydrogenation behaviour of Li_4_NH itself, however, remains essentially unknown and Li_4_NH is the only nitride hydride currently known in the Li–N–H system.


2Li_3_N + H_2_ → Li_4_NH + Li_2_NH (3)

Further, non-stoichiometric phases can be formed at 723 K from the reaction between the hydride and imide products in Equation (3). These complex non-stoichiometric phases thus contain N^3−^, H^−^ and (NH)^2−^ anions and form a solid solution [Equation (4)] [4]:

(1−*x*)Li_4_NH + *x*Li_2_NH → Li_4−2x_N_1−x_H_1−x_(NH)_x_ (4)

A full understanding of the structure and reactivity of Li_4_NH is thus required in order to determine its role in the Li–N–H system and the process of hydrogen uptake and release. One of the problems in developing such an understanding centres on the reliable synthesis of single phase Li_4_NH. Preparation of the phase requires the solid state reaction of Li_3_N and LiH at high temperature under strictly anaerobic conditions while preventing side reactions with container materials.

In this work we demonstrate how microwave synthesis of Li_4_NH using both commercial multi-mode and single-mode microwave (MW) cavities can provide a solution to this problem. The result is a reproducible route for the synthesis of phase-pure Li_4_NH over timescales orders of magnitude shorter than those required for conventional heating methods, which are less energy-efficient and more difficult to control. This facile synthesis approach has allowed us to produce bulk powders of Li_4_NH for a subsequent comprehensive study of structure, stability and reactivity. This synthesis method may well be extrapolated successfully to other hydrogen storage materials.

## 2. Experimental Section

### 2.1. Synthesis of Li_4_NH

All manipulations were performed in an N_2_-filled glovebox (5 ppm O_2_; 10 ppm H_2_O). Lithium nitride, Li_3_N (Alfa Aesar, Heysham, Lancashire, UK, 300 mesh, 99.95%; *ca.* 0.1 g) and lithium hydride, LiH (Sigma Aldrich, Gillingham, UK, 30 mesh, 95%) (1:1.1 molar ratio) were ground manually with an agate mortar and pestle, pressed into pellets (13 mm internal diameter, 1–2 mm thickness) for 30 min using a hand press and placed in an N_2_-filled silica tube (25 cm × 8 mm × 2 mm) sealed with parafilm. The silica tube was transferred from the glove box and sealed under vacuum (10^−2^ m∙bar). Reactions were conducted in either a multi-mode microwave reactor (Panasonic 4697 NN-TS53W, Panasonic UK Ltd., West Berkshire, UK, 900 Wmax. output, 2.45 GHz) or a single-mode microwave reactor (CEM Discover^®^, CEM corporation, Matthews, NC, USA, 300 Wmax. output, 2.45 GHz). The resultant products were collected in the glovebox.

### 2.2. Characterization

Powder X-ray diffraction (PXD) was conducted using a Bruker D8 diffractometer (Bruker Corporation, Billerica, MA, USA, Cu Kα source) or a PANalytical X’Pert Pro MPD powder diffractometer (Cu Kα_1_ source) in capillary mode. The air-sensitive samples were ground into fine powders and placed in 0.5 mm diameter sealed glass capillaries for data collection. Data were collected in the range 5° ≤ 2θ ≤ 85° using a 0.0168° 2θ step size for 1 h for phase identification or 10° ≤ 2θ ≤ 110° for 12 h for structure refinement. PXD data were indexed and refined by least squares fitting using the CELREF software package [6]. Structural refinements were conducted via the Rietveld method using the GSAS and EXPGUI packages [7,8] The scale factor, zero point and background were refined in initial cycles, A shifted Chebyschev polynomial function (background function 1 in GSAS) was employed to model the background. The unit cell parameters, peak profile parameters and atomic parameters were refined subsequently. The peak shape was modelled using the pseudo-Voigt function (profile function 2 in GSAS). Constraints were applied to the thermal parameters of the N and H atoms within both the LT- and HT-Li_4_NH phases.

Simultaneous thermal analysis (thermogravimetric and differential thermal analysis; TG-DTA) was performed using a NETZSCH STA 409PC thermobalance coupled to a HIDEN HPR20 mass spectrometer (MS). Approximately 30 mg of Li_4_NH was placed in an alumina pan and heated from ambient temperature to either 773 K or 873 K at 5 K∙min^−1^ under a flow of Ar or N_2_ (60 mL∙min^−1^), respectively. The maximum temperature was held for 1 h before cooling (5 K∙min^−1^). Simultaneously, mass spectra for nitrogen, hydrogen, ammonia and water were recorded during heating.

IR spectra were collected at room temperature (20 scans/sample, 8 cm^−1^ resolution) using a Shimadzu FTIR 8400S instrument with a Pike MIRacle ATR sampling accessory. Raman spectra were collected at room temperature using a Horiba LabRAM HR confocal microscope system (Horiba Itd., Kyoto, Japan) with a 532 nm laser, 1200 gr∙mm^−1^ grating and a Synapse CCD detector. A hole aperture of 50 μm and a 25 times reduced laser intensity were used in order to minimise sample decomposition.

## 3. Results and Discussion

### 3.1. Li_4_NH Synthesis Using a Multimode Microwave Reactor

MW synthesis in a commercial multimode cavity (MMC) reactor offers faster processing (over times of the order of minutes), increased energy efficiency and lower cost [9] than conventional high temperature approaches. To date, MW heating experiments with solid-state hydrogen storage materials have been limited to the study of the dehydrogenation properties of a small number of alkali, alkaline-earth and transition metal hydrides and of the alkali metal borohydrides, LiBH_4_, NaBH_4_ and KBH_4_ [10,11,12]. Nevertheless, given the difficulties in mapping the microwave field distributions in MMCs, there are some drawbacks related to this synthetic approach such as variable reaction reproducibility and poor material homogeneity.

**Figure 1:**
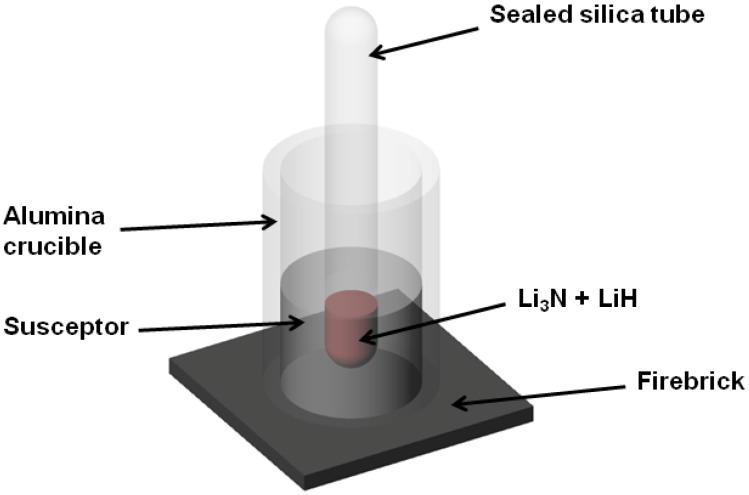
Reaction set-up using an multimode cavity (MMC) reactor.

Table 1 shows representative experiments performed to ascertain the appropriate reaction conditions for the successful synthesis of Li_4_NH (samples 1–5). A schematic of the reaction set-up for the synthesis of the samples described in this section is shown in Figure 1. Several reaction parameters such as microwave power, reaction times, atmosphere and the use of graphite (G) as an external microwave susceptor were considered. It should be noted that the reaction times in Table 1 were not continuous; each reaction was stopped at regular intervals (*ca.* 1 min steps) to allow for cooling in an attempt to avoid overheating of the silica reaction ampoule.

**Table 1:** Summary of Li_3_N + LiH reactions using an MMC reactor.

Reaction	Power/W	Time/s	Other reaction conditions	PXD results
1	900	960	Ar_(g)_ atm	LiH, α-Li_3_N
2	900	240	Vacuum	LiH, α-Li_3_N, Li_4_NH, Li_2_O and SiO_2_
3	600	70	Ar_(g)_ atm, G	LiH, α-Li_3_N and Li_4_NH
4	600	300	Vacuum, G	LiH, α-Li_3_N, Li_4_NH and Li_2_O
5	600	390	Vacuum, G	Li_4_NH, Li_2_O

**Figure 2:**
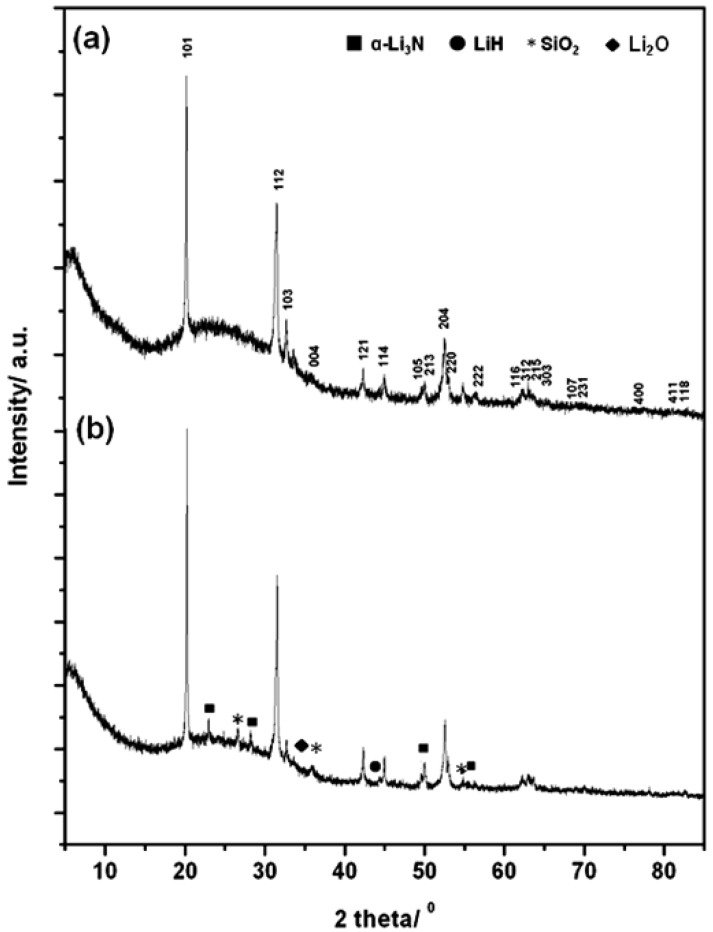
PXD data for the as-formed products obtained from MW irradiation in an MMC reactor (**a**) at 600 W for 390 s sample 5; and (**b**) at 900 W for 240 s sample 2. Indexed peaks correspond to the tetragonal Li_4_NH phase.

From these results it was shown that Li_4_NH could be synthesised under the conditions highlighted for sample 5. Figure 2 shows the PXD data obtained from sample 5; additional PXD of a partially reacted sample 2 was plotted for comparison. Silica peaks from the reaction vessel were also observed (marked with asterisks in the PXD pattern). For sample 5, the indexed cell parameters for Li_4_NH of *a* = 4.893(2) Å and *c* = 9.936(8) Å match well to those previously reported by Marx [Tetragonal space group *I*4_1_*/a*, *a* = 4.8918(1) Å, *c* = 9.9321(3) Å] [4]. Neither N–H nor O–H bands were observed in Raman spectra collected for sample 5. The use of an external microwave susceptor, *i.e.*, graphite, in these reactions appears essential in order to drive the reaction to completion. This can be rationalised in terms of the necessity to raise the reaction temperature to a point where the reactants exhibit a loss tangent (tan δ) that is sufficient to couple effectively with microwaves [13]. In fact, it has been reported that graphite achieves a temperature of 1345 K in *ca.* 2 min of microwave irradiation [13]. Reactions performed under argon gas were not observed to proceed to completion, even in the presence of a graphite susceptor.

Despite successfully preparing high purity Li_4_NH using an MMC, concerns over repeatability across experiments from uncertainties in the field distribution led us to consider the use of an single mode cavity (SMC) reactor to improve homogeneity, reproducibility and potentially further reduce reaction times. This cavity offers better control over reactions given the opportunity to accurately position the sample in a well-defined electric field [14]. Moreover, the use of an external susceptor in these reactions is avoided and therefore any risk of product contamination can be minimised.

### 3.2. Li_4_NH Synthesis Using a Single Mode Microwave Reactor

**Figure 3:**
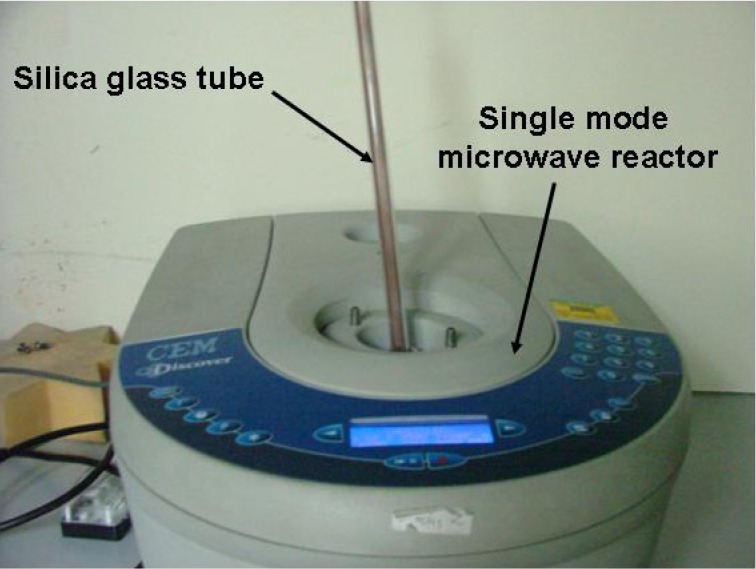
Reaction set-up using a SMC reactor.

**Figure 4:**
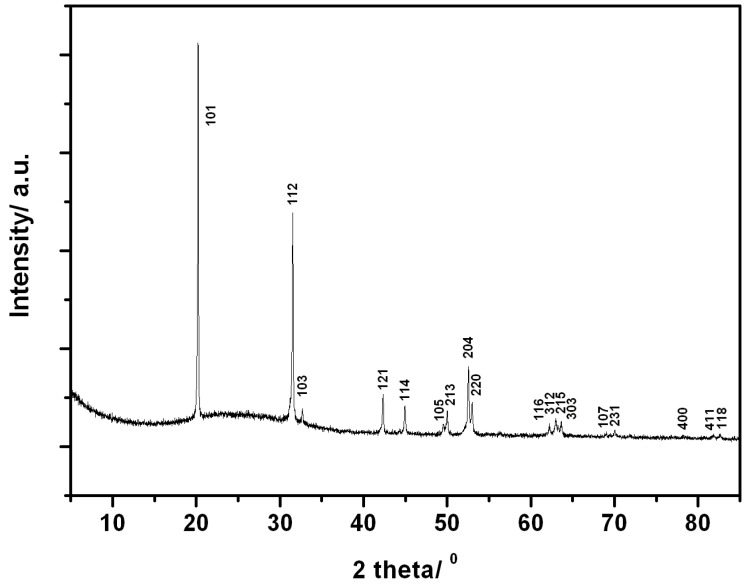
PXD data for tetragonal Li_4_NH obtained in an SMC reactor at 300 W for 180 s under vacuum (sample 8).

A summary of the reactions conducted in SMC system (Figure 3) is shown in Table 2. As for the MMC syntheses described in Section 3.1, cooling intervals were introduced between irradiation periods to avoid melting of the silica reaction ampoule (*i.e.*, melting point 2073 K). Indeed, heating at 300 W for *t* > 240 s led to the destruction of the SiO_2_ reaction vessel. It is evident from PXD data collected for sample 8 that single phase Li_4_NH could be successfully synthesized at 300 W in 180 s; no reflections from the starting materials α-Li_3_N and LiH were observed (Figure 4). The final product had the appearance of a yellow/beige pellet. Previously, Li_4_NH was synthesised from the reaction between Li_3_N and LiH at 763 K for 6 h under Ar [4] and thus with the synthetic approach described here, reaction times could be reduced by a factor of 100 and performed without the need for an inert cover gas.

**Table 2:** Summary of Li_3_N + LiH reactions using a SMC reactor.

Reaction	Power/W	Time/s	Other reaction conditions	PXD results
6	150	270	Vacuum	LiH, α-Li_3_N and Li_4_NH
7	250	270	Vacuum	LiH, α-Li_3_N and Li_4_NH
8	300	180	Vacuum	Li_4_NH

The MW synthesis of lithium nitride hydride is possible due to the ability of the starting materials to absorb microwave energy and convert this into heat (as reflected in the loss tangent, tan δ). The ability of Li_3_N to produce heat in a microwave field may be attributed to its inherent fast ionic conductivity and semiconducting behavior [15,16]. In fact, it is well established that microwaves couple directly to charge carriers leading to extremely rapid reactions in many ionic conductors and semiconductors [17]. Conversely, LiH does not generate significant heat under a microwave field and, for example, no changes in temperature were observed when LiH was placed within SMC (400 W; 20 min) or MMC (500 W; 30 min) reactors [7,9]. In fact, in these previous studies among NaH, MgH_2_, CaH_2_, TiH_2_, VH_0.81_, ZrH_2_ and LaH_2.48_ only the transition metal and lanthanide hydrides showed a rapid increase in temperature, which even then only led to the desorption of a small percentage of hydrogen (< 0.5 wt %).

During the reactions described here (samples 1–8), a purple plasma was observed along the length of the silica reaction tube. The purple plasma was followed on most occasions by yellow/orange flashes (Additional Supplementary Information). The observation of these plasmas/flashes provides evidence for the high local temperatures achieved in the reaction vessel (*i.e.*, Li evaporation occurs at *ca.* 1573 K) [18].

### 3.3. Thermal Stability of Li_4_NH 

**Figure 5:**
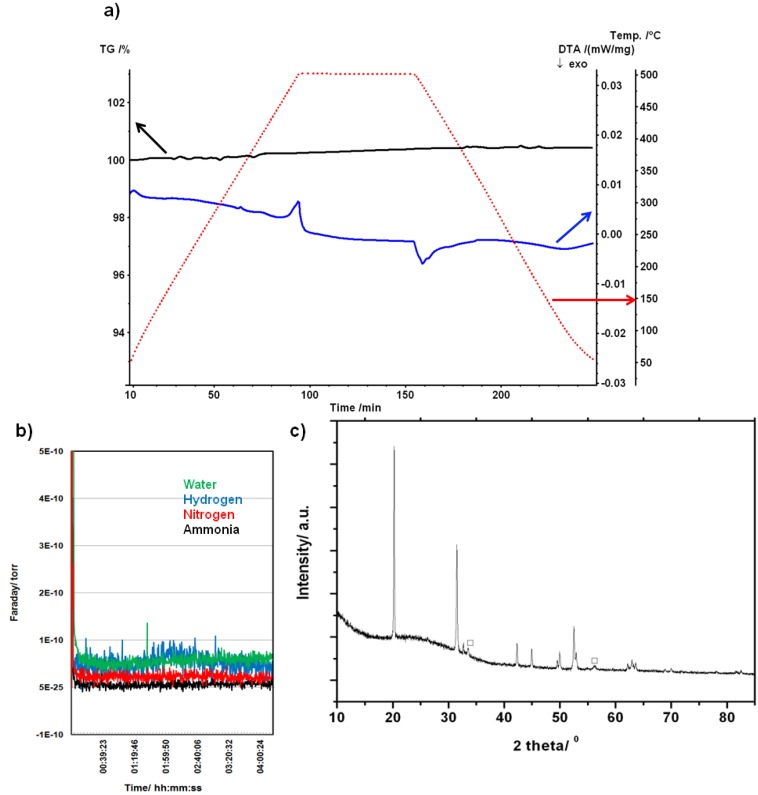
(**a**) Thermogravimetric and differential thermal analysis (TG-DTA) profiles for sample 8 under Ar_(g)_. The sample was heated to 773 K at 5 K min^−1^ held for 1 h and cooled at 5 K min^−1^. TG, DTA and temperature curves are represented in black, blue and red respectively; (**b**) Mass spectra obtained under the conditions shown in (**a**). NH_3(g)_, N_2(g)_, H_2(g)_ and H_2_O were monitored; and (**c**) PXD pattern showing the experimental data for sample 8 after heating and cooling under Ar. The open squares denote a Li_2_O minor impurity phase.

The thermal stability of the nitride hydride was investigated by TG-DTA under flowing argon. TG-DTA of sample 8 showed no evidence of mass change and hence decomposition or dehydrogenation when the sample was heated to 773 K (Figure 5a). Moreover, it was also evident from mass spectra collected simultaneously while heating that no hydrogen or other gases were evolved over the entire *m/z* range (1 ≤ *m/z* ≤ 200) (Figure 5b). These results corroborate previous investigations conducted to 698 K under argon [4]. Indexing of the PXD pattern from sample 8 following the TG-DTA experiment (Figure 5c) yielded cell parameters for Li_4_NH of *a* = 4.891(2) Å and *c* = 9.9252(8) Å. These lattice parameters are within 2σ of those obtained for this sample prior to the TG-DTA and therefore no significant changes were noted. An Li_2_O impurity was noted in the post-TG-DTA diffractogram and was attributed to the presence of moisture in the Ar_(g)_ and/or a reaction between a small amount of Li_4_NH and the alumina sample holder.

The DTA profile for sample 8 however reveals an interesting feature above 700 K with no corresponding simultaneous weight change. This endothermic peak at 770 K can thus be attributed to a structural phase transition in Li_4_NH. An equivalent exothermic peak in the DTA was observed at 755.6 K on cooling, demonstrating that the phase transition is reversible (and as corroborated by PXD where the tetragonal Li_4_NH is observed as discussed above).

**Figure 6:**
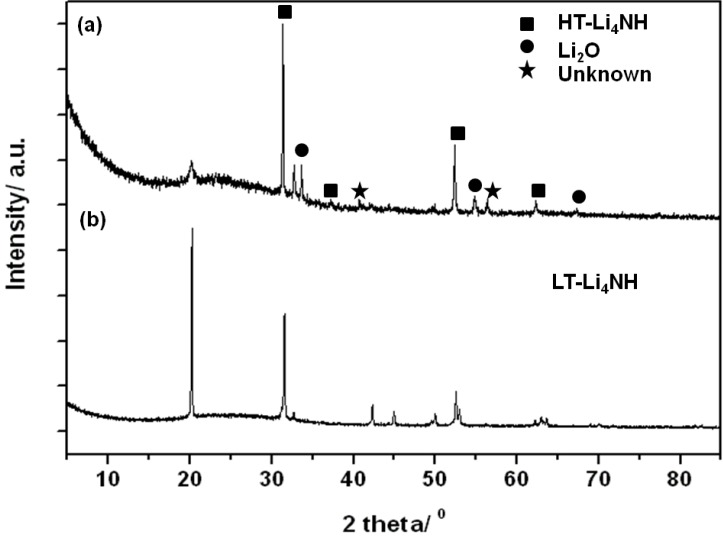
PXD pattern of sample 8 (**a**) after quenching Li_4_NH from high temperature (to form sample 9); and (**b**) prior to heating.

In light of the evidence for a high temperature phase transition from the TG-DTA data, attempts were made to isolate the high-temperature (HT) Li_4_NH phase by heating as-prepared Li_4_NH (sample 8) to 798 K at 5 K min^−1^ under flowing argon using a conventional furnace and quenching in liquid nitrogen. Figure 6 shows the PXD pattern of the reaction products collected after quenching (sample 9). The diffractogram comprises reflections for the tetragonal low temperature (LT) phase and Li_2_O but is notable for the appearance of a small number of new peaks corresponding to an HT-phase. The reflections for the HT-Li_4_NH phase could be indexed to a cube with *a* = 4.915(1) Å. The presence of Li_2_O in the reaction products is attributed to a possible reaction between Li_4_NH and the SiO_2_ reaction tube. Hence, it is quite likely that the unknown peaks observed in the powder pattern for sample 9 might correspond to Li–Si–(N–O) impurities from a side reaction with the reaction ampoule. Raman spectra collected for sample 9 showed the complete absence of either N–H or O–H bands.

### 3.4. Structure Determination

**Figure 7:**
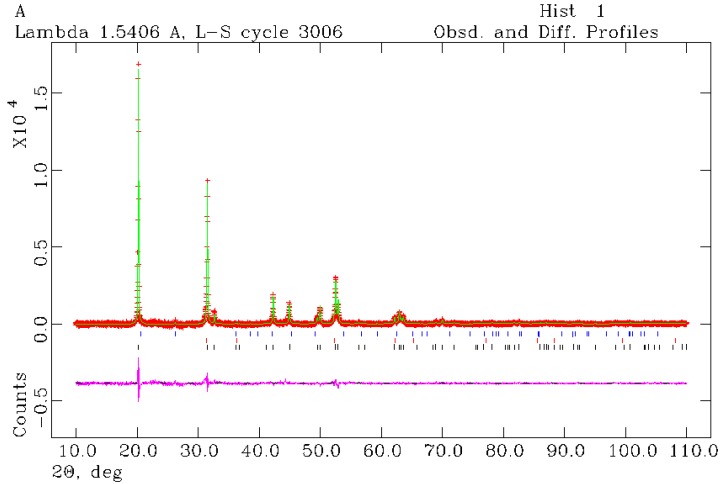
Observed-calculated-difference (OCD) profile plot from the room temperature Rietveld refinement for sample 8. Observed data are shown in red, calculated data are shown in green and the difference between the two profiles is shown in pink. Black tickmarks correspond to tetragonal Li_4_NH, red tickmarks correspond to cubic Li_4_NH and blue tickmarks correspond to SiO_2_.

Structure refinements performed against PXD data were conducted for sample 8. Selected Rietveld refinement data collected at room temperature are displayed in Table 3 and additional results are included in the Supplementary Information (Tables S1 and S2). Although a satisfactory fit for the data could be obtained using a single phase tetragonal model for LT-Li_4_NH, a marked improvement in the residuals was achieved when including the second HT-phase identified by the quenching experiments (see above). Traces of silica (assumed to originate from the reaction ampoule) were also found in the diffraction profile of sample 8. The observed-calculated-difference (OCD) profile plot is shown in Figure 7.

The presence of the HT-phase in sample 8 can be rationalised by the relatively fast cooling rate from the SMC MW reaction (as compared to conventional heating), which allows some of the kinetically stable HT-Li_4_NH phase to remain in the sample at room temperature.

**Table 3:** Selected Rietveld refinement data from the lab X-ray refinement of sample 8 at 298 K.

Empirical formula	LT-Li_4_NH	HT-Li_4_NH
Collection temperature/K	298	298
Crystal system	Tetragonal	Cubic
Space group	*I*4_1_*/a*	*Fm*$\bar{3}$*m*
Lattice parameters/Å	*a* *=* 4.8864(1)	*a* *=* 4.9462(3)
	*c* = 9.9183(2)	
*V*/Å^3^	236.82(1)	121.01(2)
*Z*	4	4
Unit cell formula weight, *M*_w_	171.116	85.558
Density/g cm^−3^	1.200	1.174
Phase fraction/wt%	98.1(5)	1.8(5)
No. of observations, parameters	12,117, 35	
*R*_wp_, *R*_p_	0.0373, 0.0273	
χ^2^	1.962	

**Figure 8:**
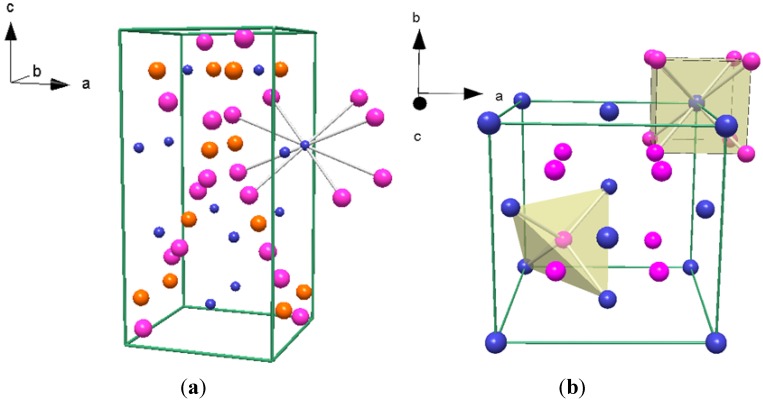
Crystal structures of (**a**) the LT-Li_4_NH phase (space group *I*4_1_*/a*), where Li atoms are represented in pink; N1/H1 are represented in orange; and N2/H2 atoms are represented in blue; and (**b**) the HT-Li_4_NH phase (space group *Fm*$\bar{3}$*m*). Lithium atoms are represented in pink and N/H atoms are represented in blue. Polyhedra showing the coordination environment of the Li and N/H atoms are represented in yellow.

The LT-Li_4_NH phase was modeled in tetragonal space group *I*4_1_*/a* with lattice parameters *a* = 4.8864(1) Å and *c* = 9.9183(2) Å (Figure 8a) and corresponds to the structure originally reported by Marx and re-determined by Niewa *et al.* [4,5].from PXD and PND data and single crystal X-ray diffraction data respectively. Initial attempts were made to fit HT-Li_4_NH in tetragonal space groups but following the elucidation of the second phase from the quenching experiments described in Section 3.3, a better structural model was chosen. The structure of HT-Li_4_NH was refined in the cubic space group *Fm* $\bar{3}$ *m* with *a* = 4.9462(3) Å (Figure 8b) using a model based on a modified Li_2_NH-type *anti*-fluorite structure in which N^3−^ and H^−^ equally occupy the 4*a* site occupied by the imido N atom in Li_2_NH [19]. The structure thus corresponds to a regular Li_2_(N,H) cube derived from the tetragonal LT-Li_4_NH structure where *a*_cubic_ ~ *a*_tetragonal_ and *a*_cubic_ ~ *c*_tetragonal_/2.

Atomic positions and isotropic thermal parameters for each Li_4_NH phase are presented in Table 4 and Table 5. Given that PXD is not able to locate hydride accurately, the occupancies of the N^3−^ and H^−^ anions in the LT-Li_4_NH phase were fixed at values reported by Marx [4]. Attempts to fix the site occupancies at alternative values representing different distributions of anions led to poorer quality fits. The LT-phase therefore exhibits anion ordering with the 4*a* site predominantly occupied by N^3−^ (at 95%) and the 4*b* site similarly almost fully occupied by H^−^. The cubic HT-Li_4_NH phase displays a disordered N^3−^/H^−^ anion distribution over the 4*a* site. The occupancies for the N^3−^ and H^−^ atoms in the HT-Li_4_NH phase were both fixed to 50% in order to achieve charge balance. Given the structural relationship between the LT- and HT-Li_4_NH phases, it is not surprising that the coordination environments around the cations and anions in each phase are very similar. Whereas in the LT- structure the essentially fully ordered N^3−^ and H^−^ anions are in a distorted cubic coordination with Li^+^ (with distances ranging between 2.044(2)–2.082(2) Å and 1.949(2)–2.556(3) Å respectively), in the HT-structure fully disordered N^3−^/H^−^ anions are in a regular cubic coordination, with an Li–(N^3−^/H^−^ distance of 2.1418(1) Å (Supplementary Information; Table S1). Li atoms are tetrahedrally coordinated to N/H atoms in the HT-phase. There are also strong similarities between the *anti*-fluorite structures of HT-Li_4_NH (Li_2_N_0.5_H_0.5_) and Li_2_NH (Li_2_(NH)). The Li–N^3−^/H^−^ bond lengths are shorter than the lithium-imido Li–N distances reported by Balogh *et al.* [19] in Li_2_NH. (2.205 Å).

Nitride hydrides are relatively rare but N^3−^/H^−^ anion ordering similar to that in the LT-Li_4_NH phase has also been observed in alkaline earth metal nitride hydrides such as Ca_2_NH(D) (cubic space group *Fd* $\bar{3}$*m*) [20,21], Ba_2_NH(D) and Sr_2_NH (both hexagonal space group *R* $\bar{3}$*m*) [22,23]. Although there are no previously reported examples of complete N^3−^/H^−^ disorder in the solid state, the anion disorder in HT-Li_4_NH is paralleled by the N^3−^/F^−^ distribution in nitride fluorides such as Ba_2_NF [24,25]. Further studies on deuterated LT-Li_4_NH and HT-Li_4_NH using powder neutron diffraction will be performed to elucidate the crystal structures more fully (*i.e.*, determine accurate hydrogen (deuterium) occupancies and anisotropic thermal parameters).

**Table 4:** Atom positions and isotropic thermal parameters generated by Rietveld refinement against lab X-ray data for LT-Li_4_NH (sample 8) at 298 K.

Atom	N1	H1	N2	H2	Li1
Site	4*a*	4*a*	4*b*	4*b*	16*f*
x	0	0	0	0	0.1959(5)
y	0.25	0.25	0.25	0.25	0.4618(4)
z	0.125	0.125	0.625	0.625	0.2794(2)
100 × U_iso_/Å^2^	3.9(1)	3.9(1)	3.1(4)	3.1(4)	6.93(9)
Site occupancy	0.95	0.05	0.05	0.95	1.00

**Table 5:** Atom positions used for Rietveld refinement against PXD data for HT-Li_4_NH (in sample 8) at 298 K. (Thermal parameters were fixed for this minority phase).

Atom	Li1	N1	H1
Site	8*c*	4*a*	4*a*
x	0.25	0	0
y	0.25	0	0
z	0.25	0	0
100 × U_iso_/Å^2^	2.5	2.5	2.5
Occupancy	1.00	0.50	0.50

### 3.5. Reactivity of Li_4_NH with Air and Nitrogen

#### 3.5.1. Li_4_NH in Air

**Figure 9:**
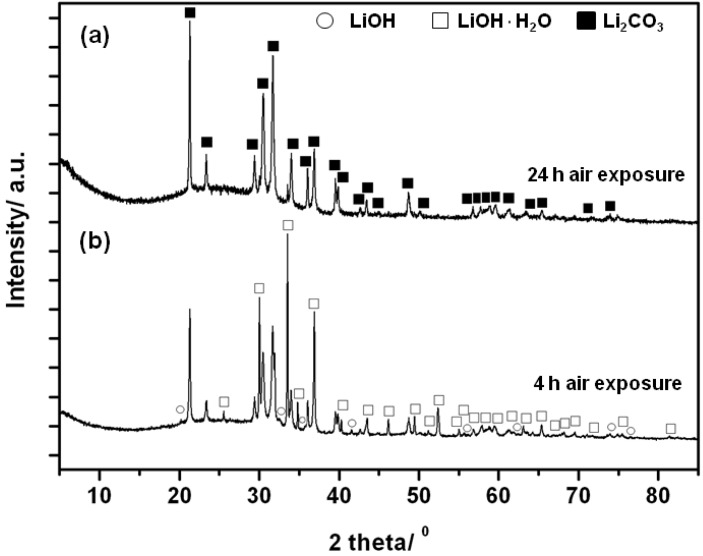
PXD patterns of the products after Li_4_NH air exposure for (**a**) 24 h; and (**b**) 4 h.

To determine the reactivity of Li_4_NH in air, a freshly made sample was exposed to the ambient atmosphere for different times and the as-formed products were analysed by PXD (Figure 9). After 4 h of air exposure, Li_4_NH had completely reacted to form crystalline phases of Li_2_CO_3_, LiOH·H_2_O and LiOH. Prolonged (e.g., 24 h) exposure of Li_4_NH to air led predominantly to Li_2_CO_3_ with some Li_2_OH·H_2_O still present. Given this experimental evidence and by analogy to Li_3_N [26], the hydrolysis of the nitride hydride is likely to proceed via LiOH formation (with evolution of ammonia and hydrogen; Equation 5) followed by further hydration to obtain the monohydrated hydroxide [Equation (6)]:

Li_4_NH + 4H_2_O → 4LiOH + NH_3_ + H_2_  ΔH_298K_= −793.117 kJ∙mol^−1^ [27] (5)

LiOH + H_2_O → LiOH·H_2_O   ΔH_298K_= −60.757 kJ∙mol^−1^ [16] (6)

Although there is the possibility that both LiOH and LiOH·H_2_O react with CO_2_ to form lithium carbonate [Equations (7) and (8) respectively], from the calculated reaction enthalpies we can establish that the former reaction is more favourable. In fact, it was not possible to identify any LiOH in the PXD pattern obtained after 24 h [although as previously stated, this also reacts with air moisture from the air to form the monohydrated LiOH (Equation 6)].


2LiOH + CO_2_ → Li_2_CO_3_ + H_2_O   ΔH_298K_ = −89.487 kJ kJ∙mol^−1^ [16] (7)

2LiOH·H_2_O + CO_2_ → 3H_2_O + Li_2_CO_3_  ΔH_298K_ = +32.027 kJ∙mol^−1^ [16] (8)

#### 3.5.2. Li_4_NH under N_2(gas)_ at Variable Temperature

**Figure 10:**
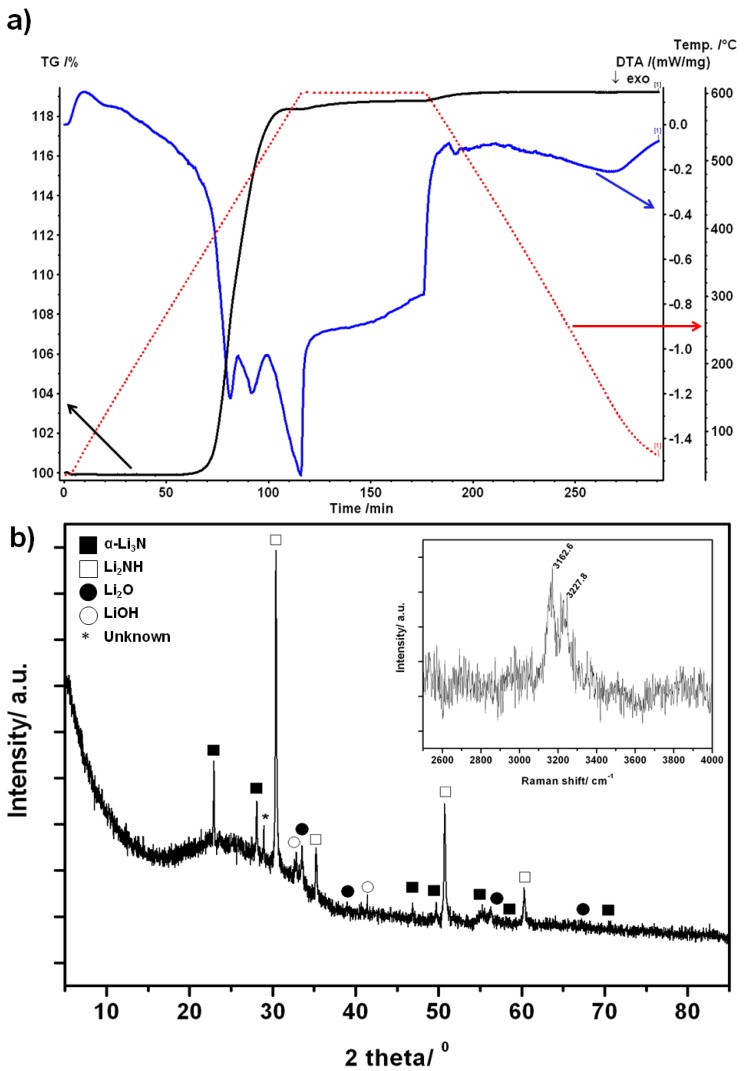
(**a**) TG-DTA plot of Li_4_NH heated to 873 K and cooled at 5 K∙min^−1^ under an N_2_ flow; and (**b**) PXD pattern of the reaction products obtained after thermal treatment under nitrogen; Inset: Raman spectra of the reaction products in the range 2500–4000 cm^−1^, showing the characteristic N-H bands from Li_2_NH.

The TG-DTA profile of the reaction between Li_4_NH and N_2(g)_ on heating to 873 K and cooling (both at 5 K∙min^−1^) (Figure 10) shows an increase in weight (with onset at *ca.* 573 K) that stabilises by 873 K. Overall, a weight gain of 19.2 wt % was achieved over the course of the reaction. Mass spectra taken during the analysis did not show any gas evolution (Supplementary Information, Figure S1). PXD of the post-STA products revealed the presence of α-Li_3_N and cubic-Li_2_NH, as the main products with no evidence of remaining nitride hydride starting material. Some evidence of LiOH and Li_2_O was also found in the PXD pattern. By contrast to spectra for Li_4_NH, further characterisation using Raman spectroscopy showed two characteristic bands at 3162.6 cm^−1^ and 3227.8 cm^−1^ (Figure 10, inset).These bands correspond to the symmetric and asymmetric N–H vibrational modes in Li_2_NH [28,29].

The mass change in the TGA profile can therefore be rationalised in terms of the reaction:

3Li_4_NH + N_2_ → 2Li_3_N + 3Li_2_NH (9)

The formation of lithium nitride and lithium imide from the nitride hydride corresponds to a theoretical gain of 21.8 wt %, which is in reasonable agreement with the experimental values obtained by thermal analysis (given also the observation of LiOH and Li_2_O as noted above). By contrast to previous suggestions, TG-DTA would thus indicate that the reaction of Li_4_NH requires a temperature in excess of 823 K to reach completion [4].

## 4. Conclusions

In summary, Li_4_NH has been synthesized in multi-mode and single-mode cavity microwave reactors over unprecedented timescales. Single-mode microwave reactions demonstrate several advantages over multi-mode approaches, such as increased efficiency and higher reproducibility. This new synthetic approach can reduce reaction times by a factor of 100 compared to conventional synthesis methods. Diffraction data served to confirm the purity of the as-formed product and to provide a structural model for Li_4_NH by means of Rietveld refinement. Thermal treatment under argon showed that a phase transition to a high temperature cubic *anti*-fluorite phase occurs at *ca.* 770 K. HT-Li_4_NH contains disordered nitride and hydride anions. In addition, results on the reactivity of Li_4_NH under air and N_2_ were also shown. In the former case, the nitride hydride reacts to form hydroxides (anhydrous and monohydrated) and subsequently lithium carbonate, under ambient conditions. In the latter case, Li_4_NH reacts to produce Li_3_N and Li_2_NH at high temperature.

## Supplementary materials

Supplementary File 1

Supplementary File 2

## References

[1] Chen P., Xiong Z., Luo J., Lin J., Tan L. (2002). Interaction of hydrogen with metal nitrides and imides. Nature.

[2] Weidner E., Bull D.J., Shabalin I.L., Keens S.G., Telling M.T.F., Ross D.K. (2007). Observation of novel phases during deuteration of lithium nitride from *in situ* neutron diffraction. Chem. Phys. Lett..

[3] Bull D.J., Weidner E., Shabalin I.L., Telling M.T.F., Jewell C.M., Gregory D.H., Ross D.K. (2010). Pressure-dependent deuterium reaction pathways in the Li–N–D system. Phys. Chem. Chem. Phys..

[4] Marx R. (1997). Reindarstellung und kristallstruktur von lithiumnitridhydrid, Li_4_NH, Li_4_ND. Z. Anorg. Allg. Chem..

[5] Niewa R., Zherebtsov A.D. (2002). Redetermination of the crystal structure of tetralithium mononitride monohydride, Li_4_NH. Z. Krist..

[6] Laugier J., Bochu B. Laboratoire des Materiaux et du Génie Physique de L’ Ecole Supérieure de Physique de Grenoble;. http://www.ccp14.ac.uk/ccp/web-mirrors/lmgp-laugier-bochu/.

[7] Larson A.C., Von Dreele R.B. (1990). Generalized Structure Analysis System (GSAS).

[8] Toby B.H. (2001). EXPGUI, a graphical user interface for GSAS. J. Appl. Crystallogr..

[9] Adam D. (2003). Microwave chemistry: Out of the kitchen. Nature.

[10] Nakamori Y., Orimo S., Tsutaoka T. (2006). Dehydriding reaction of metal hydrides and alkali borohydrides enhanced by microwave irradiation. Appl. Phys. Lett..

[11] Zhang H., Geerlings H., Lin J., Chin W.S. (2011). Rapid microwave hydrogen release from MgH_2_ and other hydrides. Int. J. Hydrog. Energy.

[12] Nakamori Y., Matsuo M., Yamada K., Tsutaoka T., Orimo S. (2007). Effects of microwave irradiation on metal hydrides and complex hydrides. J. Alloy. Compd..

[13] Rao K.J., Vaidhyanathan B., Ganguli M., Ramakrishnan P.A. (1999). Synthesis of inorganic solids using microwaves. Chem. Mater..

[14] Taylor M., Atri B.S., Minhas S., Bisht P. (2005). Developments in Microwave Chemistry.

[15] Boukamp B.A., Huggins R.A. (1978). Fast ionic conductivity in lithium nitride. Mater. Res. Bull..

[16] Gregory D.H. (2008). Lithium nitrides, imides and amides as lightweight, reversible hydrogen stores. J. Mater. Chem..

[17] Harrison A., Ibberson R., Robb G., Whittaker G., Wilson C., Youngson D. (2003). *In situ* neutron diffraction studies of single crystals and powders during microwave irradiation. Faraday Discuss..

[18] Weast R.C., Astle M.J. (1980). CRC Handbook of Chemistry and Physics.

[19] Balogh M.P., Jones C.Y., Herbst J.F., Hector L.G. Jr., Kundrat M. (2006). Crystal structures and phase transformation of deuterated lithium imide, Li_2_ND. J. Alloy. Compd..

[20] Sichla T., Jacobs H. (1995). Synthesis and crystal structure of a calcium nitride deuteride Ca_2_ND. Eur. J. Solid State Inorg. Chem..

[21] Brice J.-F., Motte J.-P., Courtois A., Protas J., Aubry J. (1976). Etude structurale de Ca_2_NH par diffraction des rayons X, diffraction des neutrons et resonance magnétique nucléaire du proton dans le solide. J. Solid State Chem..

[22] Sichla Th., Altorfer F., Hohlwein D., Reimann K., Steube M., Wrzesinski J., Jacobs H. (1997). Kristallstrukturbestimmung an einer strontium-hydrid-imid-nitrid-phase-Sr_2_(H)N/SrNH bzw. Sr2(D)N/SrND-mit röntgen-, neutronen-und synchrotron-strahlung. Z. Anorg. Allg. Chem..

[23] Altorfer F., Bührer W., Winkler B., Coddens G., Essmann R., Jacobs H. (1994). H^−^-jump diffusion in barium-nitride-hydride Ba_2_NH. Solid State Ion..

[24] Bailey A.S., Hughes R.W., Hubberstey P., Ritter C., Smith R.I., Gregory D.H. (2011). New ternary and quaternary barium nitride halides; synthesis and crystal chemistry. Inorg. Chem..

[25] Seibel H., Wagner T.R. (2004). Preparation and crystal structure of Ba_2_NF. J. Solid State Chem..

[26] Gregory D.H. (2001). Nitride chemistry of the s-block elements. Coord. Chem. Rev..

[27] Crivello J.-C., Gupta M., Černý R., Latroche M., Chandra D. (2010). Density functional study of Li_4_NH and Li_1.5_NH_1.5_ as intermediary compounds during hydrogenation of Li_3_N. Phys. Rev..

[28] Kojima Y., Kawai Y. (2005). IR characterizations of lithium imide and amide. J. Alloy. Compd..

[29] Varin R.A., Jang M., Polanski M. (2010). The effects of ball milling and molar ratio of LiH on the hydrogen storage properties of nanocrystalline lithium amide and lithium hydride (LiNH_2_ + LiH) system. J. Alloy. Compd..

