# Supplementary material for: Rapid Microwave Synthesis, Characterization and Reactivity of Lithium Nitride Hydride, Li4NH
Source: Materials (Basel). 2013 Nov 21;6(11):5410–26. doi: 10.3390/ma6115410 (PMC5452770; doi:10.3390/ma6115410)
Supplement: Supplementary File 1 [file materials-06-05410-s001.pdf]

Supplementary Information

Figure S1. Mass spectra obtained under the conditions shown in Figure 10 (**a**) $N_{2(g)}$; and (**b**) H_2O, $NH_{3(g)}$, and $H_{2(g)}$ were monitored.

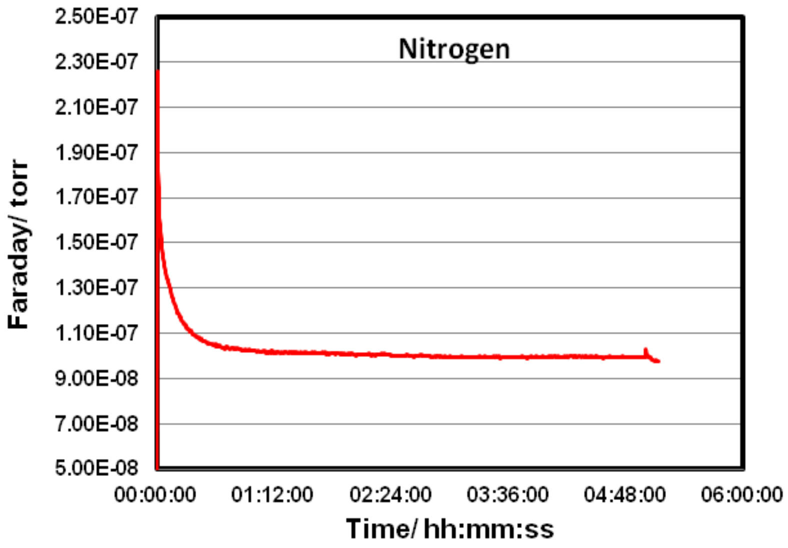

(**a**)

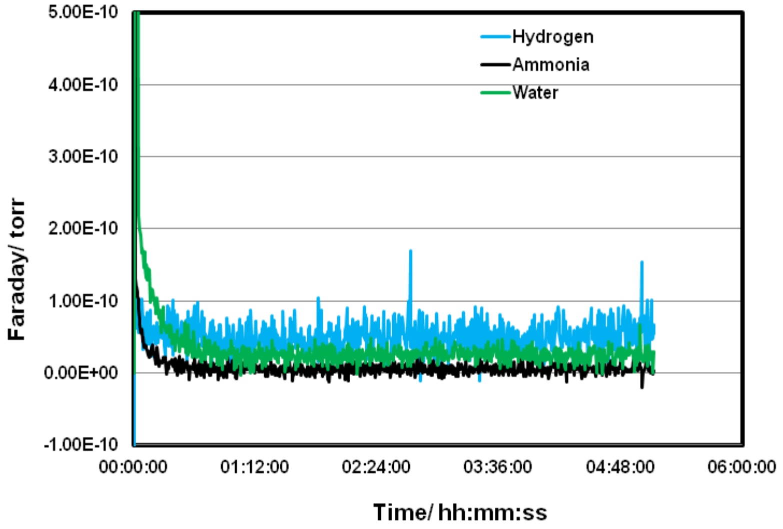

(**b**)

Table S1. Interatomic distances in Li_4NH (LT and HT phase) obtained from Rietveld refinement against PXD data at 298K.

**Interatomic distances/Å**	**LT-Li_4NH (tetragonal)**	**HT-Li_4NH (Cubic)**
Li1-(N1,H1)	4 × 2.082(2)<br>4 × 2.044(2)	2.1418(1)
Li1-(N2,H2)	4 × 1.949(2)<br>4 × 2.556(3)	

Table S2. Bond angles in Li_4NH (LT and HT phases) obtained from Rietveld refinement against PXD data collected at 298 K.

**Bond angles/ °**	**LT-Li_4NH phase (tetragonal)**	**HT-Li_4NH phase (cubic)**
Li1-(N1,H1)-Li1	4 × 75.00(5)<br>4 × 75.34(5)<br>4 × 122.76(8)<br>4 × 159.3(1)<br>2 × 85.3(1)<br>4 × 64.93(8)<br>4 × 102.43(4)<br>1 × 124.7(1)	3 × 54.736(3)<br>3 × 125.264(3)
Li1-(N2,H2)-Li1	4 × 103.69(6)<br>2 × 121.8(1)	

Additional Supplementary Information:

Short video of SMC MW reaction in progress—experiment.avi.
